# Preparation of a Water-Based Photoreactive Azosulphonate-Doped Poly(Vinyl Alcohol) and the Investigation of Its UV Response

**DOI:** 10.3390/polym11010169

**Published:** 2019-01-18

**Authors:** Philipp Nothdurft, Jörg Guido Schauberger, Gisbert Riess, Wolfgang Kern

**Affiliations:** Chair in Chemistry of Polymeric Materials, Montanuniversitaet Leoben, Otto-Glöckel-Straße 2, A-8700 Leoben, Austria; phino@gmx.net (P.N.); jgschauberger@gmail.com (J.G.S.); wolfgang.kern@unileoben.ac.at (W.K.)

**Keywords:** coatings, crosslinking, photochemistry, poly(vinylalcohol), azosulphonate, photoresist

## Abstract

Two different azosulphonate dyes were synthesised and purified for the preparation of a water-based photoreactive azosulphonate-doped poly(vinyl alcohol). The aim was the investigation of a novel azosulphonate-poly(vinyl alcohol) photoresist with decreased water solubility after illumination, setting a focus on environmentally benign substances. The electron distribution of the aromatic rings of the two different azosulphonate molecules were changed by the UV-induced cleavage of the –N=N–SO_3_^−^ groups, which was evidenced by UV spectroscopy. The formation of ester groups was detected by Fourier-transform infrared and ^13^C nuclear magnetic resonance spectroscopy. UV–Vis spectroscopy was used to investigate the photoreactivity of the prepared films. Photolithographic experiments demonstrated the applicability of these newly produced materials as photoresist materials. In addition, these materials provide high thermal stability.

## 1. Introduction

In recent decades, environmental compliance has come to be of growing importance in industry and for the application of chemical technologies and materials. Hence, production processes have to be adapted, solvents and process chemicals have to be recirculated, and the overall ecological footprint should be reduced. The substitution of established processes, chemicals and materials by water-based systems would pose alternatives that are environmentally benign, as well as leading to cost reductions after implementation. 

For example, modern photoresist materials are, in general, formulated in organic solvents, developed in strong alkaline aqueous solutions, and used for the production of circuit boards, flat panel displays, television screens and semiconductor devices [1,2]. In recent years, the development of water-based photoresist has gained more and more attention [3,4,5,6]. The advantage of this system is that it can simplify the imaging process while being more environmentally desirable.

One potential candidate for a water-based photoresist material is poly(vinyl alcohol) (PVA). PVA is usually prepared by polymer-analogous saponification of poly(vinyl acetate) which has been polymerised by free radical polymerisation [7]. Nowadays, PVA is employed as an adhesive for packaging applications [8], a sizing agent for paper processing [9,10], a water-soluble glue and modelling material [8], an encapsulation agent, and a dispersing aid [11]. Due to its biocompatibility, this polymer is regarded as safe for coatings in contact with foodstuff and suitable for the preparation of hydrogels for biomedical applications [12,13]. 

PVA is highly polar and exhibits strong hygroscopy. Thus, the formation of crosslinks between the polymer chains would lead to a reduction of the water solubility. Poly(vinyl alcohol) can be crosslinked by acid-catalysation methods or by radiation [14,15,16,17]. Exposure to ionising radiation such as β/γ-rays or X-rays results in a statistic formation of free radicals and their subsequent recombination [18]. A more convenient method to crosslink PVA is doping with transition metal chlorides such as iron chloride, or chromates such as potassium dichromate, which leads to photosensitising of the polymer. The mechanism of crosslinking can be compared to the well-known gelatine/dichromate systems in the field of photographic and holographic recording materials. It has to be stated that chromates are highly toxic compounds, which are hazardous to the environment and are carcinogenic as well [19].

Manivannan, Kuncser and Filoti investigated the mechanism of crosslinking for Fe^3+^-doped PVA and found that photoreduction of the Fe^3+^ ions to Fe^2+^ takes place, which leads to radical formation and recombination, thus leading to crosslink formation [17,20]. As FeCl_3_ is employed as flocculation agent for waste water treatment, it is considered environmentally benign and poses an alternative to chromate doping of PVA [17,20]. Schauberger took up and continued the idea of iron doping of PVA [16,21]. Here, Fe^2+^ was intercalated in montmorillonite and PVA/montmorillonite composites were UV-irradiated. Crosslinking was evidenced, and as a result, the gel content increased to 90%.

Another approach to obtain UV-curable PVA is the grafting of copolymers with acrylic moieties attached to the polymer backbone. By the addition of a radical photoinitiator such as 2-hydroxy-2-methylpropiophenone (Irgacure® 1173; BASF GmbH; Ludwigshafen; Germany), UV-induced crosslinking via free radical reaction can be performed, which leads to a material that is insoluble in deionised water [22].

**Figure 1:**
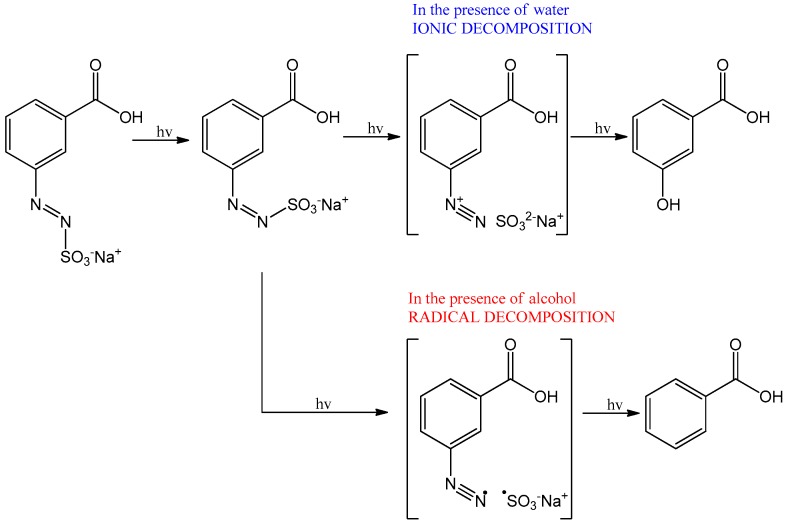
Decomposition mechanism of aryl azosulphonate compounds dependent on the chemical environment, as published by Nuyken, Voit and Riess (copyright permission by Riess) [23,26].

In this paper, the focus was set on covalently bound azosulphonates as potential crosslinking agents. It was shown by Overberger and Nuyken that thermally cleavable azo compounds can be used as initiators for free radical polymerisation [23,24]. In the early 1990s, Riess, Nuyken and Voit [23,25,26,27] investigated photoactive aryl azosulphonate dyes for the preparation of UV-active resins and polymers and studied their decomposition behaviour. It was found that the chemical environment is responsible for the decomposition mechanism (Figure 1).

Generally, the presence of water causes an ionic scission mechanism, which leads to the formation of hydroxyphenyl groups. In contrast to this, phenyl groups are formed due to the radical cleavage mechanism, caused by the presence of alcohols. The UV-induced decomposition leads to the formation of nitrogen gas, showing no dependence on the chemical environment [23,26,28]. Today, photolabile and thermally low molecular aromatic azo compounds are employed in the field of photoresist materials, laser ablation and recording materials [26,29,30].

The aim of this work is to provide a novel PVA photoresist material with decreased water solubility after illumination, based on environmentally benign materials and reaction conditions.

## 2. Materials and Methods 

### 2.1. Materials

3-amino isophthalic acid, 3-amino benzoic acid, sodium sulphite, sodium carbonate and sodium nitrite were obtained from Sigma-Aldrich Co. (Vienna, Austria), Hydrochlorid acid was obtained from Carl Roth GmbH Co. KG (Karlsruhe, Germany). These chemicals were used without any further purification or particular treatment. PVA was provided by DuPont Chemical Company (Lenzing, Austria) and characterised prior to use.

### 2.2. Methods

#### 2.2.1. Synthesis of the Azosulphonate Sodium Salts

For the synthesis of 3,5-dicarboxylphenyl azosulphonate sodium salt (AZOII), 200 mmol of 3-amino isophthalic acid was dissolved in 100 mL of hydrochloric acid solution (10 wt % in deionised water) and cooled to 0 °C in an ice bath. Diazotation took place by reaction of the amino groups with 100 mL of a sodium nitrite solution (2 M). Furthermore, the suspension was slowly added into a solution of sodium sulphite (240 mmol) and sodium carbonate (300 mmol) at 0 °C while stirring continuously.

The resultant orange mixture reaction was stored in a refrigerator at 0–5 °C for 18 h. Afterwards, the pH was adjusted by adding concentrated hydrochloric acid until a value of pH 2 was reached. The resulting precipitate was filtered, dried at 50 °C and ground in an agate mortar to achieve a fine powder. The product was purified by recrystallisation from warm water.

The synthesis of 3-carboxyphenyl azosulphonate sodium salt (AZOIII) was performed in a similar way. As precursor material, 3-amino benzoic acid was used, and the amount of the solution comprising sodium sulphite and sodium carbonate was reduced to 200 and 150 mmol, respectively. All other reaction steps were kept the same.

#### 2.2.2. Photolysis

The light-induced decomposition reactions of the azosulphonate sodium salts (Figure 1) were investigated via FTIR and UV–Vis spectroscopy. Therefore, aqueous solutions of AZOII and AZOIII compounds were prepared.

For FTIR spectroscopy, CaF_2_ platelets were coated with a thin layer of the respective azosulphonate salt, irradiated for a period of time (0–900 s), and transmission spectra in the range of 1800–850 cm^−1^ were recorded. The main focus was on Ar–N (1300–1250 cm^−1^), –N=N– (1260–1235 cm^−1^) and –N–S– (1055 cm^−1^) absorption bands due to decomposition during illumination. In addition, the possible formation of hydroxyphenyl groups due to the presence of aerial humidity was investigated. FTIR spectra were recorded with a PerkinElmer Spectrum One instrument (PerkinElmer Inc., Waltham, MA, USA). 

Changes in the electron density of the aromatic ring at different irradiation times (0–210 s) were monitored by UV–Vis spectroscopy. The absorption spectra were recorded in the spectral range from 200 to 800 nm in a quartz cuvette. UV–Vis spectra were taken with an Agilent Cary 50 instrument (Agilent Technologies, Santa Clara, CA, USA). 

The irradiation was conducted by using a spot curing device (high pressure mercury vapour UV light source) with a power output of 9.25 mW∙cm^−2^.

#### 2.2.3. Thermolysis

The thermal stability and the decomposition temperature of the azosulphonate salts were investigated via thermogravimetric measurements (TGA). Samples were placed in aluminium oxide crucibles and analysed. Nitrogen was used as purge gas with a flow rate of 30 mL∙min^−1^ and the measurement was performed at a heating rate of 20 K∙min^−1^. A PerkinElmer Pyris 7 (PerkinElmer Inc., Waltham, MA, USA) was used for these measurements. 

#### 2.2.4. Preparation of Azosulphonate-Doped PVA

PVA was characterised prior to use by means of gel-permeation chromatography (GPC) and ^1^H-NMR spectroscopy. GPC was performed at the Department of Physical and Theoretical Chemistry at the University of Graz; NMR spectra were recorded with a 400 MHz Agilent NMR spectrometer (Santa Clara, United States). A polydispersity index of 1.32 and a Mw of 130,000 g/mol were determined, and the specified degree of saponification (>99 mol %) was confirmed.

The aqueous PVA solution with a solid content of 5 wt % was obtained by addition of PVA to deionised water under stirring. After 10 min, the slurry was heated to a temperature of 85 °C for at least 60 min to obtain a homogeneous solution. 

The respective azosulphonate-doped PVAs (PAII and PAIII) were prepared as follow: The appropriate amount of the azosulphonate salt (Table 1) was dissolved in the 5 wt % aqueous PVA solution at a pH value of 3 adjusted with hydrochloric acid. For a better distribution of the azosulphonates, the samples were treated by sonication and stirred at 60 °C. The solutions were stored at room temperature in brown glass vials to prevent light-induced decomposition of the PVA-AZO compounds.

#### 2.2.5. PVA-AZO Thin Film Preparation

The gravity settling method was used for preparing thin films (GS films). Therefore, 10 g of the solution was poured into polystyrene petri dishes with a diameter of 85 mm, and dried under constant air flow at room temperature. Thin films were also produced by drop casting on CaF_2_ disks (DC films). A drop of the respective PVA-AZO solution was placed on the CaF_2_ crystal, and the solvent was evaporated. 

#### 2.2.6. Annealing

The prepared GS films, as well as the coated CaF_2_ platelets, were heat-treated at 100 °C using a circulating air oven for 5 to 60 min. FTIR spectra were recorded of the thin DC films prior and after annealing to determine the anticipated formation of ester bonds. In addition, the films were irradiated with a high-pressure mercury vapour UV light 0–300 s. and the reaction kinetics were monitored by UV–Vis spectroscopy.

Additionally, sample PAII.5 and sample PAIII.5 (see Table 1) were analysed by ^13^C NMR measurements. Non-heat-treated and heat-treated thin films (annealed at 100 °C for 60 min) were dissolved in deuterated water (D_2_O) at 85 °C. The changes in the NMR spectra were monitored.

#### 2.2.7. Photolithographic Patterning

Photolithographic patterning was performed by placing a quartz/chromium mask with 100-micron features onto thin PVA-AZO films, followed by illumination with a medium-pressure mercury vapour emitter with an intensity of 740 mW·cm^−2^ for 60 s. To enhance the visualisation of the pattern caused by decomposition of the azo dyes, a combination of phase contrast imaging and polarised light imaging was employed. Optical micrographs were recorded after patterning and after development in deionised water.

## 3. Results and Discussion

### 3.1. Characterisation of the Azosulphonate Sodium Salts

**Figure 2:**
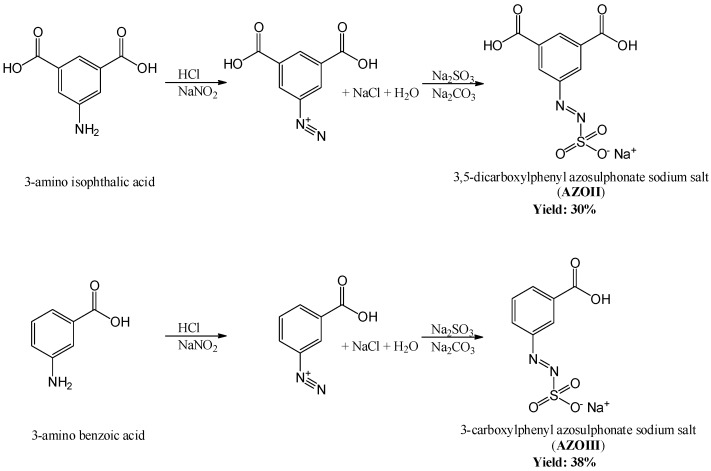
Two-step synthesis of water-soluble aryl azosulphonates.

The two-step synthesis of water-soluble aryl azosulphonates bearing carboxylic moieties on the aromatic ring is shown in Figure 2.

**Figure 3:**
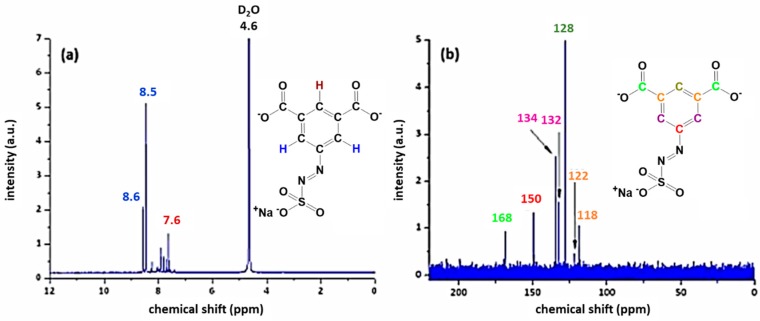
^1^H (**a**) and ^13^C (**b**) NMR spectra of AZOII dissolved in deuterated water.

**Figure 4:**
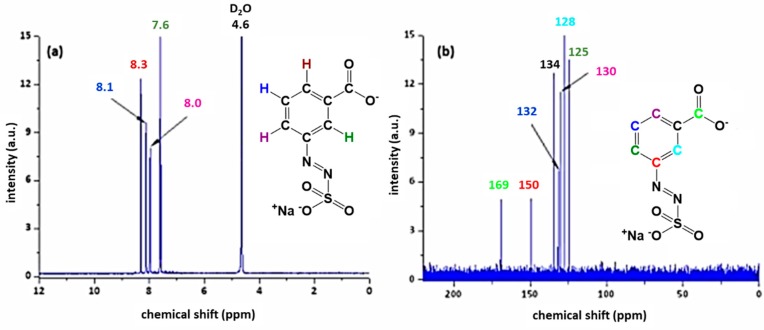
^1^H (**a**) and ^13^C (**b**) NMR spectra of AZOIII dissolved in deuterated water.

A reaction yield of 30% (60 mmol) for AZOII and 38% (76 mmol) for AZOIII was calculated. The obtained azosulphonate dyes were dissolved in deuterated water (D2O) followed by ^1^H and ^13^C NMR measurements to evaluate the products, as well as their purity. The spectra for AZOII and AZOIII are depicted in Figure 3 and Figure 4, respectively.

The protons of the aromatic ring of AZOII can be assigned to the peaks at 8.6 and 8.5 ppm (meta position, Figure 3a). The remaining hydrogen atom is therefore related to the peak at 7.6 ppm. As additional chemical shifts between 7.6 and 8.5 ppm are detected, it is assumed that these additional peaks are a result of partial deprotonation of the carboxylic moieties, by changing the dipole moment of the molecules [26,31].

The ^13^C NMR spectrum of AZOII is not affected by the deprotonation, each of the carbon atoms can be assigned to a specific peak. The carboxylic acid groups can be attributed to the peak at 169 ppm, while the –C–N=N–SO_3_^−^ shift is detected at 150 ppm. The remaining five carbon atoms of the aromatic ring can be assigned to the chemical shifts between 134 and 118 ppm and are colour coded in Figure 3b.

In accordance with the results for AZOII, the ^1^H NMR spectrum of AZOIII (Figure 4a) reveals the deprotonation of the carboxylic moiety of the azo dye; otherwise, a signal at a chemical shift of 11 ppm would be detectable (signal of the COOH proton). The other hydrogen atoms of the aromatic system are unambiguously assigned to a specific chemical shift whereby the change of the dipole moment by only one carboxylic moiety is not as strong as for the AZOII dye.

The ^13^C NMR spectrum of AZOIII exhibits a strong signal of the carboxylic moiety at 169 ppm, as well as the Ar–N=N–SO_3_^−^ shift at 150 ppm. The position of the carbon atoms of the aromatic ring is colour coded in the range of 134 to 125 ppm (see Figure 4b).

From these results, the high purity of the synthesised azosulphonate dyes was evaluated. The dyes were used for the subsequent experiments.

### 3.2. Behaviour of the Synthesised Azosulphonate Dyes under Thermal Load and UV Light

Photolysis and thermolysis experiments were performed to investigate the thermal stability and the UV sensitivity of both newly synthesised molecules.

**Figure 5:**
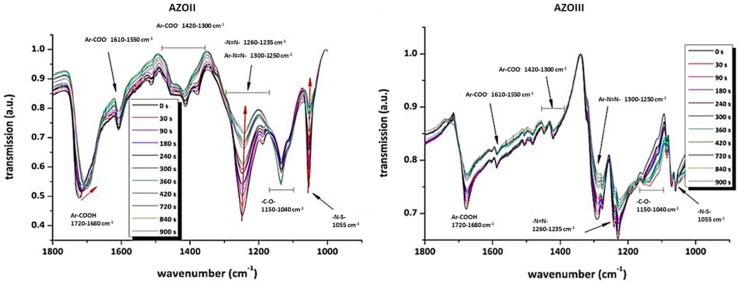
FTIR analysis of the AZOII (**a**) and AZOIII (**b**) dye on CaF_2_ platelets at different illumination times.

The carboxylic moieties of the AZOII and AZOIII dyes are partly deprotonated, which is observable by the formation of absorption bands at 1610–1550 and 1420–1300 cm^−1^, as well as by the change of the strong signal of the –COOH groups at 1720–1680 cm^−1^ (see Figure 5). This is a result of the acidification to pH 2 in the precipitation step, where a residue of carboxylates is left. This is found for both azo dyes [31,32,33].

Irradiation leads to a decrease of the Ar–N=N– and –N=N– stretching vibrations from 1300 to 1235 cm^−1^, as well as the –N–S– stretching vibration at 1055 cm^−1^. As the para substituent of the aromatic systems is decomposed, the dipole moment of the whole molecule is changed, which also explains the shift of the Ar–COOH vibration for AZOII from 1720 cm^−1^ to lower wavenumbers. Additionally, a slight increase of the –C–O– valence vibration at 1150 to 1040 cm^−1^ is observed, which may be a result of hydroxyphenyl formation, according to Nuyken and Voit (compare Figure 1) [23]. As a result of UV irradiation, the azo groups of both azo dyes are decomposed, which leads to a nitrogen gas release and splitting off the sulphonic acid moiety. 

This proposed decomposition mechanism is confirmed by the obtained FTIR kinetics and can also be followed by UV–Vis measurements, as the electron density of the aromatic system is altered due to UV-induced decomposition of the chromophore.

**Figure 6:**
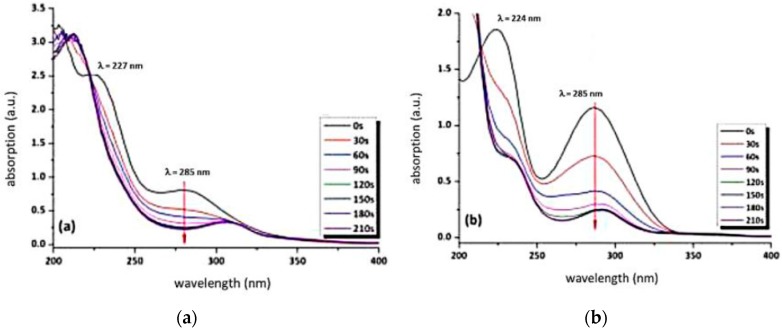
UV–Vis absorption spectra of aqueous solutions of (**a**) AZOII and (**b**) AZOIII upon exposure to UV light.

The change in the UV–Vis absorption spectra at wavelengths of 224 and 227 nm of both azo dyes can be assigned to changes in the electron density of the aromatic system as a result of UV-induced decomposition (see Figure 6a,b). As photoinduced decomposition takes place, a depletion of the absorption at 285 nm is observed (Figure 6a,b). This peak is attributed to the π–π* transition. The n-π* transition of the chromophore azosulphonate group should occur around a wavelength of 400 nm; however, due to the small absorption coefficient, it is not discernible [23,25,29].

**Figure 7:**
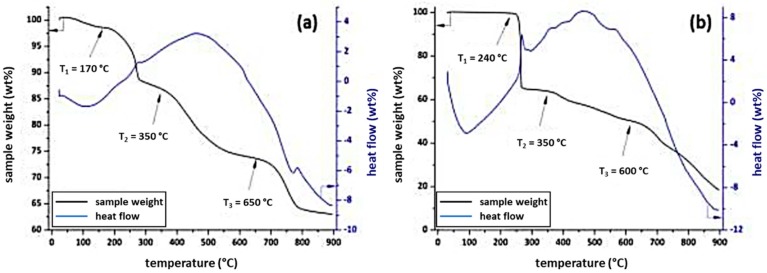
TGA curves (weight loss in relation to the initial sample weight) and the corresponding heat flow of (**a**) AZOII and (**b**) AZOIII.

As the 3,5-dicarboxyphenyl azosulphonate sodium salt exhibits high hygroscopy, it takes up aerial water which can be observed as a mass loss of 2.5 wt % up to 150 °C in the thermogravimetric curve, as well as an endothermic heat flow (see Figure 7a). The monoacid AZOIII, on the other hand, does not exhibit such behaviour, but it displays a higher thermal stability, with the decomposition starting at 240 °C, while AZOII shows an onset of decomposition at 170 °C (Figure 7a,b).

This first decomposition temperature can be related to fragmentation of the azo groups, which leads to nitrogen gas emission [34]. The cleavage is very rapid for AZOIII, with a first sample weight loss of about 37 wt %, while AZOII shows a weight loss of 25 wt % (compare Figure 7a,b). The first decomposition step is followed by decarboxylation and the decomposition of the aromatic ring, which leads to a further mass loss of 15 wt % for AZOII and of 10 wt % for AZOIII26. Finally, thermal decomposition of the residual sulphonic acid groups takes place at temperatures ranging up to 800 °C for AZOII and 750 °C for AZOIII [35]. In general, AZOII is thermally more stable, with an ash content of around 60 wt %, whereas the overall weight loss for AZOIII (>80 wt %) leads to the conclusion that the decomposition products of this substance are more volatile.

### 3.3. Characterisation of Azosulphonate-Doped PVA

**Figure 8:**
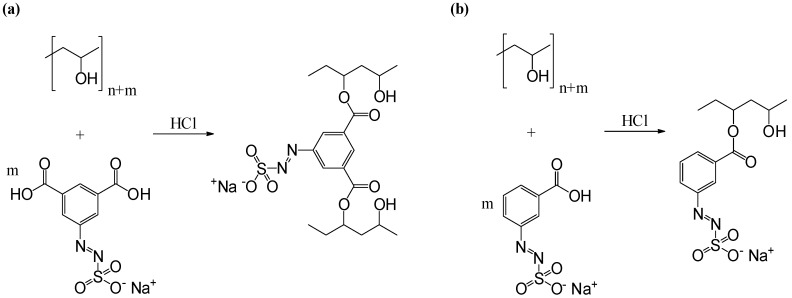
Mechanism of coupling reaction of aryl azosulphonates to PVA via polymer analogous esterification reaction: (**a**) crosslinking of PVA by coupling of AZOII; (**b**) coupling of monovalent AZOIII to PVA.

Condensation reactions and radical polymerisation enhance the crosslink density in azosulphonate-doped PVA, which could reduce the hydrophilicity and solubility of neat PVA [36]. The resulting AZO-doped PVA can be used as photoresist, where the illuminated regions are more water resistant than the non-illuminated parts, which are partly dissolved after development in water. Therefore the azosulphonates AZOII and AZOIII were used to dope poly(vinyl alcohol) to achieve a coupling between the polymeric chains (with AZOIII) or an increase in crosslinking density (with AZOII) by radical crosslinking during UV illumination. In Figure 8, the reaction to covalently link the azo dyes to PVA is shown.

The amount of the two azosulphonates in PVA was varied, and for these formulations, thin films were produced, which were subjected to further treatment (UV, heat). 

**Figure 9:**
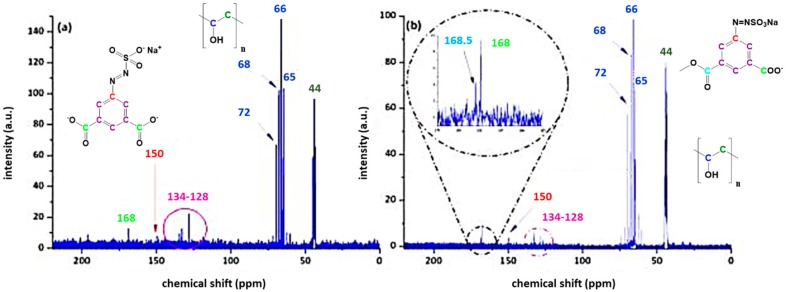
^13^C NMR of PAII.5 (comprising 20 wt % of AZOII) before (**a**) and after (**b**) annealing at 100 °C for 60 min.

Sample PAII.5 without heat treatment gives a superposition of the ^13^C NMR spectra of the source materials (see Figure 9a). It is evident that the detection of specific C atoms reaches the detection limit of the measurement system; however, it is possible to determine the structural elements, which are colour coded in Figure 9. Upon heat treatment, the formation of aromatic ester linkages can be evidenced by the splitting of the carbonyl shift at 168 ppm into two different signals (see detail in Figure 9b) [31]. One signal is attributed to carboxylic acid, the other to ester units.

**Figure 10:**
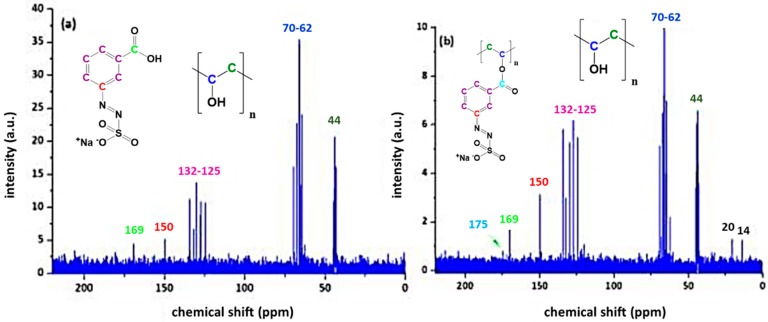
^13^C NMR of PAIII.5 (comprising 20 wt % of AZOII) before (**a**) and after (**b**) annealing at 100°C for 60 min.

The coupling reaction of the monovalent acid AZOIII can also be followed by ^13^C NMR spectroscopy and is similar to the NMR results of AZOII doped PVA (Figure 9). The evolution of a second carbonyl signal at 175 ppm (see Figure 10b) is also attributed to an aromatic ester formation, while the signal at 169 ppm stems from non-converted carboxylic groups [37]. As these signals are in general very faint, additional FTIR measurements were conducted on thin films to ascertain these findings.

**Figure 11:**
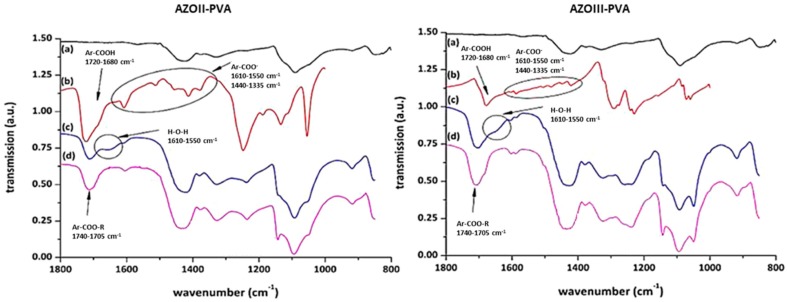
FTIR spectra of AZOII-PVA (**left**) and AZOIII-PVA (**right**) polymers; (**a**) neat PVA; (**b**) the respective AZO dye; (**c**) PAII.5/PAIII.5 containing 20 wt % of AZO dye; (**d**) PVA-AZO after 60 min of annealing at 100 °C.

The carboxylic groups of the AZO dyes are partially deprotonated for the pristine azosulphonates, as well as for the doped PVA films and the heat-treated samples. The formation of aromatic ester groups by condensation reaction of PVA and the azo dye is indicated by the shift of C=O vibrations at around 1700 cm^−1^ to lower (AZOII) and higher wavenumbers (AZOIII) (Figure 11a–d) [31,32]. In addition, the appearance of a band prior to and after annealing at 1150 cm^−1^ is assigned to C–O–C bonds supporting ester formation. Despite these findings, the occurrence of the condensation reaction is uncertain. The assigned ester and carboxylic bands overlap, and the C–O–C vibration is in the same wavenumber region as the C–O vibrations of carboxylic acid dimers. As a result, the reaction is not clearly evidenced.

**Figure 12:**
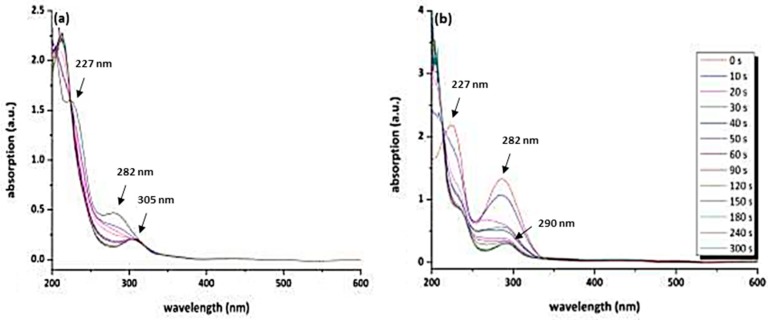
UV–Vis absorption spectra (**a**) of annealed PAII.5 and (**b**) of annealed thin PAIII.5 films coated onto a CaF_2_ substrate.

After the annealing step, investigations of the photolysis kinetics were performed by UV–Vis spectroscopy. In the following section, the photolysis behaviour of PAII.5 and PAIII.5 is illustrated and discussed (Figure 12). 

UV–Vis spectroscopy is appropriate to investigate UV-induced decomposition of the azo dye in the doped PVA films. As the electron density of the aromatic ring is altered by the cleavage of the azo group, the absorption at 227 nm is diminished and shifted towards lower wavelengths for both azo dyes [26,30,38]. The second absorption maximum at 282 nm (attributed to the π–π* transition) decreases as well. This leads to a bathochromic shift of the absorption to 305 nm for PAII.5 and to 290 nm for PAIII.5.

### 3.4. Photolithographic Patterning

**Figure 13:**
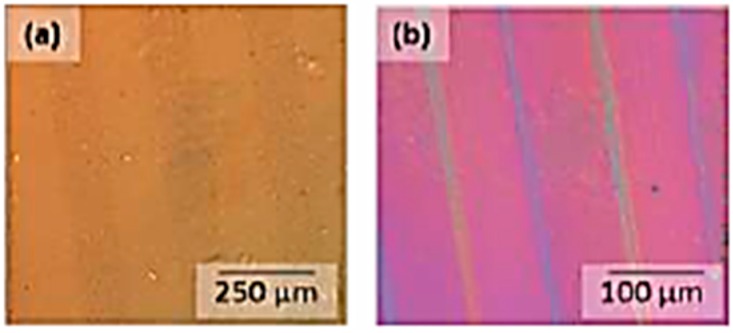
Phase contrast image of thin patterned PAII.1 films (**a**) and after development in deionised water (**b**).

**Figure 14:**
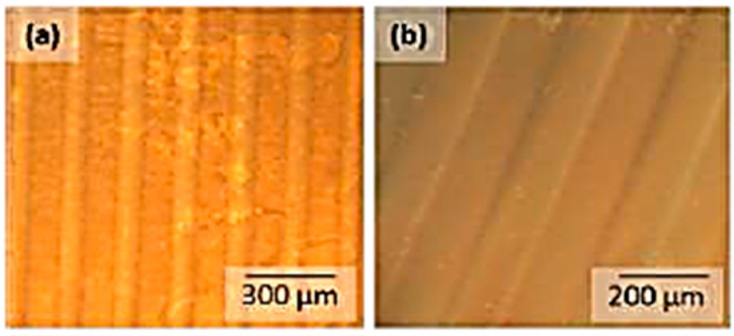
Phase contrast image of thin patterned PAIII.1 films (**a**) and after development in deionised water (**b**).

The addition of comparatively small amounts of AZO dyes (2.5 wt %) leads to well-resolved linear patterns with sharp boundaries, which give topographical features after development. In general, the photoactivity for PVA films containing the carboxylic acid AZOIII is way better compared to AZOII-doped PVA films and leads to a good contrast behaviour of the patterned samples (see Figure 13 and Figure 14). The crosslinking of PVA chains by radical decomposition of the covalently bound AZO dye plays a more important role in PVA-AZOIII thin films than in AZOII-doped PVA, where crosslinking had already occurred during annealing by condensation reactions. It is also observed that with increasing dye content, the number of surface defects increases after development, which is caused by outgassing of cleavage products (nitrogen gas). 

**Figure 15:**
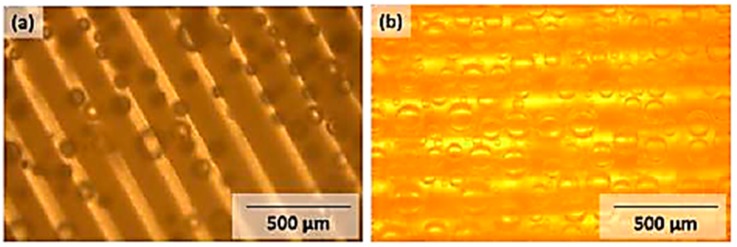
Gas bubble formation upon the development of patterned samples (100 μm features; dose: 45 Jcm^−2^) in deionised water using phase contrast imaging mode. (**a**) PAII.5; (**b**) PAIII.5.

Samples containing 20 wt % of photoactive species appear grainy, which may be caused by phase separation during the drying step. During development of such samples (PAIII.5), extensive gas bubble formation occurs after a short time in deionised water (see Figure 15b).

The obtained structures are a result of decomposition of the chromophore azo groups and the release of nitrogen gas. During the development step, gas bubble formation, most likely nitrogen gas resulting from UV-induced cleavage of the azosulphonate dyes, takes place. The bubbles are formed in the illuminated areas (bright lines, see Figure 15), thus giving further evidence that the patterning step is the origin of this effect.

## 4. Conclusions

Water-soluble aryl azosulphonate dyes with carboxylic moieties attached to the aromatic ring (AZOII/ZOIII) were prepared in accordance to published work [23,26]. The photolytic behaviours of AZOII and AZOIII were investigated by decomposition in aqueous solution, providing information that the electron density of the aromatic ring is changed by UV-induced cleavage of the –N=N–SO_3_^−^ group. These results are confirmed by FTIR spectroscopy of thin films on CaF_2_ platelets, by the depletion of the –C–N=N– and –N=N– absorption bands, as well as by the –N–S– absorption. Thermolysis experiments demonstrate the high-temperature stability of the prepared photoactive dyes, with decomposition temperatures higher than 170 °C for the dicarboxylic acid AZOII, and 240 °C for AZOIII.

In a novel approach, the prepared aryl azosulphonate compounds were covalently attached to poly(vinyl alcohol). At first, the coupling between the photoactive carboxylic acids and the polymer by heat-induced esterification was studied. Due to detection limit issues, it is difficult to prove the coupling reaction of the carboxylic moieties and the hydroxyl groups of the polymer by means of FTIR and ^13^C NMR spectroscopy. UV–Vis spectroscopy was employed due to its enhanced sensitivity to visualise UV-induced changes in the prepared materials. As neat PVA exhibits no UV absorption, changes in the absorption spectra result from photolytic cleavage of the azosulphonate units, which can be attributed to the π–π* transition, whereas the n-π* transition is not discernible due to the low absorption coefficient.

These novel UV-reactive materials exhibit good contrast behaviour after photolithographic patterning, which can be visualised by polarised light and phase contrast imaging. This leads to the conclusion that changes of the optical contrast of the thin films are a result of the cleavage of the azosulphonate groups. For samples containing a high content (20 wt %) of azo sulphonate, strong gas bubble formation is observed during the development of the illuminated samples.

Due to the thermal stability of both the azosulphonate compounds and PVA, applications for high-temperature coatings with tuneable polarity depending on the chemical environment would be possible, e.g., waveguides, optical components. Further adaptions concerning the coupling process of the azosulphonates onto the polymer backbone could lead to full conversion of the carboxylic moieties. 

## Figures and Tables

**Table 1:** Composition of PVA-AZO formulations (with regard to the dry content).

Notation	PVA (wt %)	AZOII (wt %)	AZOIII (wt %)
PAII.1	97.5	2.5	
PAII.2	95	5	
PAII.3	92.5	7.5	
PAII.4	90	10	
PAII.5	80	20	
PAIII.1	97.5		2.5
PAIII.2	95		5
PAIII.3	92.5		7.5
PAIII.4	90		10
